# *Dictyostelium discoideum* as a non‐mammalian biomedical model

**DOI:** 10.1111/1751-7915.13692

**Published:** 2020-10-30

**Authors:** Javier Martín‐González, Javier‐Fernando Montero‐Bullón, Jesus Lacal

**Affiliations:** ^1^ Molecular Genetics of Human Diseases Group Department of Microbiology and Genetics Faculty of Biology University of Salamanca Campus Miguel de Unamuno Salamanca E‐37007 Spain; ^2^ Metabolic Engineering Group Department of Microbiology and Genetics University of Salamanca Campus Miguel de Unamuno Salamanca E‐37007 Spain

## Abstract

*Dictyostelium discoideum* is one of eight non‐mammalian model organisms recognized by the National Institute of Health for the study of human pathology. The use of this slime mould is possible owing to similarities in cell structure, behaviour and intracellular signalling with mammalian cells. Its haploid set of chromosomes completely sequenced amenable to genetic manipulation, its unique and short life cycle with unicellular and multicellular stages, and phenotypic richness encoding many human orthologues, make *Dictyostelium* a representative and simple model organism to unveil cellular processes in human disease. *Dictyostelium* studies within the biomedical field have provided fundamental knowledge in the areas of bacterial infection, immune cell chemotaxis, autophagy/phagocytosis and mitochondrial and neurological disorders. Consequently, *Dictyostelium* has been used to the development of related pharmacological treatments. Herein, we review the utilization of *Dictyostelium* as a model organism in biomedicine.

## Graphical abstract

This MS opens a window to the general audience on the potential of *Dictyostelium* as a model microbe in biomedicine.

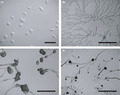

## Introduction

The cellular slime mould *Dictyostelium discoideum* is a protist that has long been regarded as a valuable and attractive tool for the study of eukaryotic cell biology because a high number of conserved functions and host‐pathogen interactions comparable to human cells (Annesley and Fisher, 2009). *Dictyostelium* provides a potential valuable vehicle for studying functions of protein human orthologues in a system which is experimentally tractable with an intermediate complexity between yeasts and higher multicellular eukaryotes (Eichinger *et al*., 2005). Based on studies that survey the presence of human orthologues in *D. Discoideum*, probing a set of genes related to human disease, the number of hits was estimated highly relevant (22%) and similar to other model organisms such as *D. melanogaster* or *C. elegans*, while higher than in *S. cerevisiae* or *S. pombe* (Eichinger *et al*., 2005). *Dictyostelium* has a 34 Mb haploid genome with six chromosomes encoding ~ 12 500 proteins (Steinert and Heuner, 2005). Its genome has been entirely sequenced and detailed genomic and proteomic information can be found in dictyBase (http://dictybase.org/) (Kreppel *et al*., 2004; Chisholm, 2006; Fey *et al*., 2009, 2013; Gaudet *et al*., 2011; Basu *et al*., 2013). In particular, *Dictyostelium* is one of eight non‐mammalian model organisms recognized by the National Institute of Health (NIH) in the United States for its utility in the study of fundamental molecular processes of human medical importance (Goldberg *et al*., 2006).

**Fig. 1:**
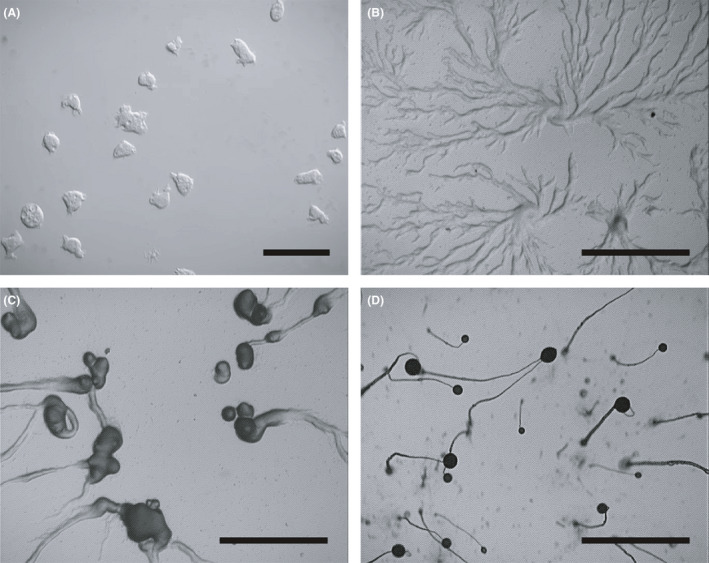
*Dictyostelium* cells (A) and developmental time course (B, C, D) in the absence of nutrients. Wild‐type AX3 cells grown in shaking axenic culture were plated on glass (A), and on non‐nutrient agar plates (B, C, D) and allowed to starve (*t* = 0 h) as previously described (Lacal *et al*., 2018). (A) Image of AX3 cells plated on glass by digital interference contrast microscopy. In the absence of nutrients starvation is imminent, the amoebae stop dividing and activate several genes that will allow them to aggregate by chemotaxis towards cAMP diffusing from centrally located cells. Pictures of developing cells were taken during (B) aggregation (*t* = 6 h), (C) mound (*t* = 12 h) and (D) after completion of formation of fruiting bodies (*t* = 24 h) using time‐lapse phase‐contrast microscopy. Scale bar in (A), 50 µm, whereas in (B, C, D) represents 1 mm.

Its developmental life cycle is unique among protists and at the different stages of development, *Dictyostelium* features both plant‐ and animal‐like characteristics (Barth *et al*., 2007). Different stages in the life cycle of *D. discoideum* are shown in Figure 1. *Dictyostelium* grows by the mitotic division of single cells that feed by phagocytosis on bacteria, or by macropinocytosis on simple axenic liquid medium, making it possible to reach high cell densities (Williams *et al*., 2006; Paschke *et al*., 2019). Upon starvation, *Dictyostelium* cells exhibit an impressive multicellular cooperativity and start to aggregate by chemotaxis in response to released cAMP signals (Steinert and Heuner, 2005). More than 100,000 cells are forming the aggregate called motile slug. The slug responds thermotactically and phototactically with exquisite sensitivity (Annesley and Fisher, 2009). After the formation of a motile slug, the differentiation culminates in the production of a fruiting body consisting of 80% spore cells and 20% dead stalk cells (Steinert and Heuner, 2005).

*D. discoideum* has been studied for many years, and some papers have presented it as a relevant model in biomedicine (Hägele *et al*., 2000; Steinert and Heuner, 2005; Chisholm, 2006; Alibaud *et al*., 2008; Annesley and Fisher, 2009; Bozzaro and Eichinger, 2011; Francione *et al*., 2011; Terbach *et al*., 2011; Bozzaro, 2013; Tatischeff, 2013; Annesley *et al*., 2014; Cunliffe *et al*., 2015; Otto *et al*., 2016; Frej *et al*., 2017; Mesquita *et al*., 2017; Domínguez‐Martín *et al*., 2018; Leoni *et al*., 2018; McLaren *et al*., 2019; Pearce *et al*., 2019; Schaf *et al*., 2019; Tatischeff, 2019; Thewes *et al*., 2019; Perry *et al*., 2020). To the best of our knowledge, *D. discoideum* was not just first isolated but also studied within the biomedical field in the infection of human pathogen bacteria (Raper and Smith, 1939). However, *Dictyostelium* will not be considered as a biomedical model until many years later (Saxe, 1999). Nowadays, recent papers have a different and wider perspective from those of the beginning, from studies of the extracellular vesicle (EV) in cancer (Tatischeff, 2013, 2019), the endoplasmic reticulum stress (Domínguez‐Martín *et al*., 2018) or microbiome regulation and homeostasis in humans (Farinholt *et al*., 2019), to CRISPR technology applications (Iriki *et al*., 2019). In this minireview, we recapitulate the major areas using *D. discoideum* as a model organism in biomedicine.

## Infection by bacterial pathogens

**Fig. 2:**
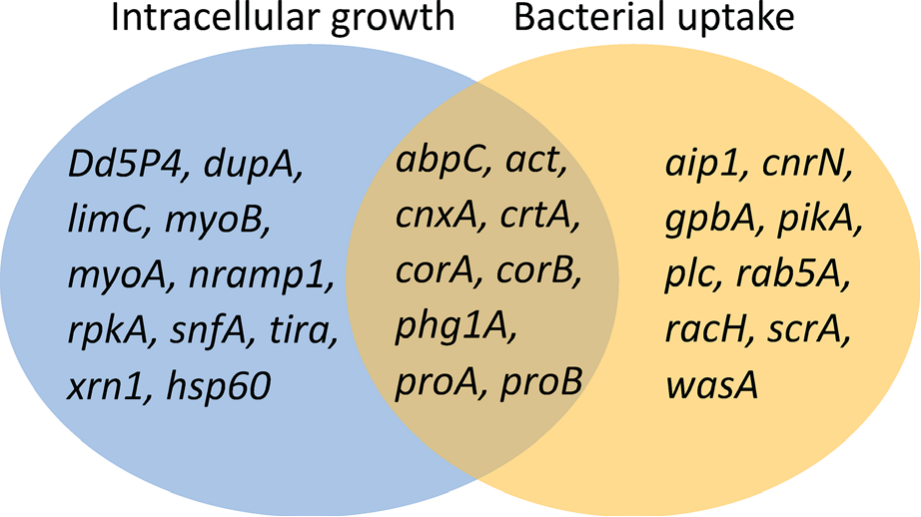
*D. discoideum* genes with implications in pathogenesis. A total of 29 genes have been identified in *Dictyostelium* as host model for pathogenesis. The encoded proteins are involved in intracellular growth (blue), bacterial uptake (yellow) and in both processes (overlapping area).

Due to the similarities between *D. discoideum* and human cells, *Dictyostelium* is a good model for the study of microbial infection since the damage made by the pathogen is mimicked (Annesley and Fisher, 2009). *Legionella pneumophila* is perhaps the most studied bacterial pathogen in *Dictyostelium*. During the colonization of the human respiratory tract, *L. pneumophila* enters and multiplies within alveolar macrophages, leading to severe pneumonia called Legionnaires disease (Hägele *et al*., 2000; Williams *et al*., 2006). In order to characterize the intracellular life cycle of Legionella, investigators have used a variety of host cells, including free‐living protozoa and human cells (Hägele *et al*., 2000). In human cells *L. pneumophila* enters in the macrophages by phagocytosis, then recruits endoplasmic reticulum vesicles where it starts to multiply, and newly form bacteria get out of the host by lysis (Williams *et al*., 2006; Annesley and Fisher, 2009). *D. discoideum* has been used to analyse the uptake, the dynamics movements of *L. pneumophila* containing vacuole (LCV), the transcriptional changes after infection, the protein composition of LCV and also to study some of the *Legionella* virulence factors and cellular targets (Bozzaro *et al*., 2019). The most studied proteins are related to the uptake process, which is made by conventional phagocytosis, including but not limited to G proteins (*gpbA*), phospholipase C (*plc*), calnexin (*cnxA*), calreticulin (*crtA*) and cytoskeleton‐associated proteins (Fig. 2) (Steinert and Heuner, 2005; Williams *et al*., 2006). Regarding the uptake and bacterial replication in the phagosome, both processes have been studied in *D. discoideum* profilin‐minus (well‐conserved actin‐binding proteins) strains, that are more susceptible of *Legionella* infection (Fajardo *et al*., 2004). On the other hand, Nramp1‐minus (orthologue of the mammalian SLC11a1) *Dictyostelium* cells displayed a reduced phagocytosis and a higher intracellular growth of *L. pneumophila* because depletes the phagosome of iron (Steinert and Heuner, 2005; Bozzaro and Eichinger, 2011). *Dictyostelium* Ca^2+^‐binding proteins with chaperone activity in the endoplasmic reticulum calnexin and calreticulin were found to be involved not only in endocytosis but also in exocytosis (Williams *et al*., 2006).

Apart from the uptake via phagocytosis, there are evidences that suggest macropinocytosis as another mechanism for bacteria uptake (Bozzaro and Eichinger, 2011). Some other factors related with both, phagocytosis and macropinocytosis, are Arp2/3 complex, RpkA (rpkA), WASP (*wasA*) and WAVE (*scrA*), small G proteins of the Rho family (such as Rab5, Rab7, Rab8 and Rab14, which are necessary for the fusion of phagosome with lysosome) and actin‐binding proteins such as coronin (*corA*), a well‐conserved protein in *Dictyostelium* (Thewes *et al*., 2019) whose absence produces a reduced *Legionella* uptake and enhances intracellular growth (Leoni Swart *et al*., 2018). All the above proteins are essential in the uptake of *L. pneumophila* (Annesley and Fisher, 2009; Bozzaro and Eichinger, 2011) (Fig. 2). Rac proteins (Bozzaro and Eichinger, 2011) and PTEN (*pten*) (Annesley and Fisher, 2009; Bozzaro *et al*., 2019) are also necessary for the uptake of nutrients. The decrease in PI(4,5)P₂ has been related with a higher *Legionella* infection in collaboration with PIPLC (PIPLC inhibitors do not let phagocytosis to happen), PI3K (*pikA*) (inhibition of PI3K would let a higher *Legionella* replication) and the PI‐5‐phosphatase (*Dd5P4*), helping the fusion of vesicle and lysosome (Bozzaro and Eichinger, 2011).

Besides the discoveries made in the uptake process, *Dictyostelium* helped identifying new host cell factors for intracellular growth including LimC/LimD (*limC*), myosin I (*myoA and myoB*), profilin (*proA* and *proB*) and Nramp1 (*nramp1*) (Hägele *et al*., 2000) (Fig. 2). Also, the presence of inositol polyphosphate 5‐phosphatase (Dd5P4), similar to the human protein OCRL1, reduces pathogen replication and the LCV formation (Bozzaro and Eichinger, 2011). AMP‐activated protein kinase (AMPK) (*snfA*) overexpression, a central cellular energy sensor, helps for a higher proliferation of *L. pneumophila* (Annesley and Fisher, 2009; Bozzaro and Eichinger, 2011). Further, a defective AMPK in *D. discoideum* causes reduced growth, impaired aggregation, misdirection and mislocalization at the slug stage, impaired slug phototaxis and thermotaxis (Annesley and Fisher, 2009).

Last but not least, we would like to mention that there are more pathogens for which *D. discoideum* was used as a model organism to study infection. *Bordetella* genus is involved in pathologies such as whooping cough. Recently, species of the genus *B. bronchiseptica* were proposed to use *Dictyostelium* as an environmental reservoir (Taylor‐Mulneix *et al*., 2017). Other pathogens include but are not limited to *Mycobacterium marinum* (Hagedorn *et al*., 2009)*, Salmonella typhimurium* (Sillo *et al*., 2011)*, Pseudomonas aeruginosa* (Cosson *et al*., 2002; Pukatzki *et al*., 2002; Alibaud *et al*., 2008)*, Klebsiella pneumoniae* (Lima *et al*., 2014)*, Francisella noatunensis subsp. Noatunensis* (Brenz *et al*., 2018)*, Cryptococcus neoformans* (Steenbergen *et al*., 2003) and yeast such as *Candida albicans* (Koller *et al*., 2016). These organisms are less studied than *L. pneumoniae*, but they allowed the identification of key proteins including the orthologue of the mammalian SLC11a1 protein (encode by *Dictyostelium nramp1*, which depletes iron from the phagolysosome in an ATP‐dependent process), myosin (*myoA* and *myoB*, involved in cell motility) and atg1 (*atg1*, serine/threonine protein kinase involved in autophagy). These and other host key proteins in *D. Discoideum* have been unravelled (Table 1, Table S1).

**Table 1:** *D. discoideum* proteins involved in *Legionella pneumophila* infection. M.m.: *Mycobacterium marinum*, S.m.: *Salmonella typhimurium*, K.p.: *Klebsiella pneumoniae*, F.n.: *Francisella noatunensis subsp. Noatunensis*, C.n.: *Cryptococcus neoformans*, C.a.: *Candida albicans*.

Gene name (*Dictyostelium*)	UniProt *(Dictyostelium*)	Species	Gene name (*Dictyostelium*)	UniProt *(Dictyostelium*)	Species
*abpC*	P13466	*L.p*.	*wasA*	Q9GSG9	*L.p*.
*act*	P07830	*L.p*.	*xrn1,hsp60*	Q75JF5,Q54J97	*L.p*.
*aip1*	P54686	*L.p*.	*atg1*	Q86CS2	*F.n/ C.a,*.
*cnrN*	Q54JL7	*L.p*.	*Kil1*	Q55GK8	*C.a,*
*cnxA, crtA*	Q55BA8,Q23858	*L.p*.	*fspa*	Q86K54	*K.p*.
*corB*	Q55E54	*L.p*.	*rtoA*	P54681	*C.n*.
*Dd5P4*	Q8I7P3	*L.p*.	*Rd1*	C7G076	*M.m*.
*dupA*	Q550K8	*L.p*.	*csaA*	P08796	*S.t.*.
*gpbA*	P36408	*L.p*.	*csbA*	P16642	*S.t*.
*limC*	Q9BIW5	*L.p*.	*csbC*	Q558X5	*S.t*.
*myoB, myoA*	P34092,P22467	*L.p./ C.n*.	*cadA*	P54657	*S.t*.
*nramp1*	Q869V1	*L.p./ F.n*	*cad2*	O97113	*S.t*.
*phg1A*	Q55FP0	*L.p*.	*dscA‐a*	P02886	*S.t*.
*pikA*	P54673	*L.p*.	*dscE*	P42530	*S.t*.
*plc*	Q02158	*L.p*.	*dscC‐1*	P02887	*S.t*.
*proA, proB*	P26199,P26200	*L.p*.	*dicB*	Q55GS3	*S.t*.
*rab5A*	Q86JP3	*L.p*.	*cbpA*	P35085	*S.t*.
*RacH*	Q9GPR7	*L.p*.	*cbpC*	P54653	*S.t*.
*rpkA*	Q86D86	*L.p*.	*cbpD1*	Q54RF4	*S.t*.
*snfA*	Q54YF2	*L.p*.	*cbpG*	Q54QT8	*S.t*.
*tirA*	Q54HT1	*L.p*.			

## Directed migration, or chemotaxis, of immune cells

Many immune cells can detect the direction and intensity of an extracellular chemical gradient and migrate towards the source of stimulus. This process, called chemotaxis, is essential for immune system function and homeostasis (Mañes *et al*., 2005). As aforementioned, some studies on *D. discoideum* exploit the similarities with macrophages in the uptake of pathogens such as *Legionella*. In *Dictyostelium*, vegetative cells access nutrient sources by migration towards products such as folic acid derived from bacteria or yeast, or locally secreted cAMP in the formation of motile slug during periods of starvation (Artemenko *et al*., 2014) (Fig. 1). This amoeboid movement is well conserved along eukaryotic evolution and resembles movement in human cells such as leucocytes or metastatic tumour cells (Artemenko *et al*., 2014). Indeed, many immunity diseases are linked with defects in leucocyte and macrophage chemotaxis and can also be modelled in *Dictyostelium* (Carnell and Insall, 2011). Interestingly, there are many similarities between the chemotactic signalling pathway of *Dictyostelium* and leucocytes, where G‐protein‐coupled receptors (GPCRs) signal changes cytoskeletal dynamics (Artemenko *et al*., 2014). Regarding immunity diseases, Wiskott–Aldrich syndrome is caused by mutations in the *WAS* gene and is characterized by abnormal or non‐functional white blood cells (Carnell and Insall, 2011). *D. discoideum* WASP protein (*wasA*) contributes to front‐rear cell polarity by controlling localization and cellular levels of activated Rac (*rac1B*, *racA* and *racG*) (Amato *et al*., 2019). Shwachman–Diamond syndrome particularly affects the bone marrow, pancreas and bones. This syndrome is caused by mutations in the *SBDS* gene encoding a protein that is required for the assembly of mature ribosomes and ribosome biogenesis. *Dictyostelium* SBDS localizes to the pseudopodia in cAMP gradient (Williams *et al*., 2006), and when mutated, caused defective PMN leucocytes orientation towards a *N*‐formylmethionyl‐leucyl‐phenylalanine (fMLP) spatial gradient (Artemenko *et al*., 2014). Many other *Dictyostelium* proteins involved in chemotaxis are conserved in humans, such as TORC2 (formed by the *tor*, *lst8*, *rip3* and *piaA* products) (Annesley and Fisher, 2009), RAS (*rasC*, *rasD* or *rasG*) (Carnell and Insall, 2011) PTEN (*pten*) and PI3K (*pikA*) (Annesley and Fisher, 2009), PKB (*pkbA*) and PAKa (*pakA*) (Annesley and Fisher, 2009) (Table 2, Table S2). Also *Dictyostelium* has allowed to study the chemotaxis in tumour cells, by the GPCRs signalling pathways mentioned above, and also by the LEGI model, in which receptor occupancy by the chemoattractant triggers a fast, local excitatory signal and a lower global inhibitory signal (Roussos *et al*., 2011).

**Table 2:** *D. discoideum* proteins related to directed cell migration of immune cells. Proteins are classified by its biological function including actin cytoskeleton organization, regulation of signal transduction, protein phosphorylation and small GTPase‐mediated signal transduction.

Gene name (*Dictyostelium*)	UniProt (*Dictyostelium*)
Actin cytoskeleton organization	
*alxA*	Q8T7K0
*cosA*	Q558Y7
*DDB_G0284937*	Q54NX5
*gnrC*	Q551I6
*lst8*	Q54D08
*pakA*	Q55D99
*piaA*	O77203
*pikA*	P54673
*pkbA*	P54644
*ripA*	C7G030
*scrA*	Q54NF8
Protein phosphorylation	
*DDB_G0293184*	Q54C77
*pakC*	Q55GV3
*pakD*	Q55DD4
Small GTPase‐mediated signal transduction	
*carA‐1*	P13773
*gemA*	Q55G45
*gflD*	Q54WL2
*kxcA*	Q54GY6
*kxcB*	Q54C71
*rac1B*	P34145
*racA*	P34147
*racG*	Q9GPS0
*rasC*	P32253
*rasD*	P03967
*rasG*	P15064
*xacA*	Q54DW4
Small GTPase‐mediated signal transduction	
*gefC*	Q8IS20
*gxcC*	Q54P24
*gxcCC*	Q54XA7
*gxcD*	Q55G27
*gxcT*	Q55DL8
*pten*	Q8T9S7
*raptor*	Q55BR7
*roco10*	Q6XHA6
*roco5*	Q1ZXD6
*roco9*	Q6XHA7
*tor*	Q86C65

## Neurological disorders

**Fig. 3:**
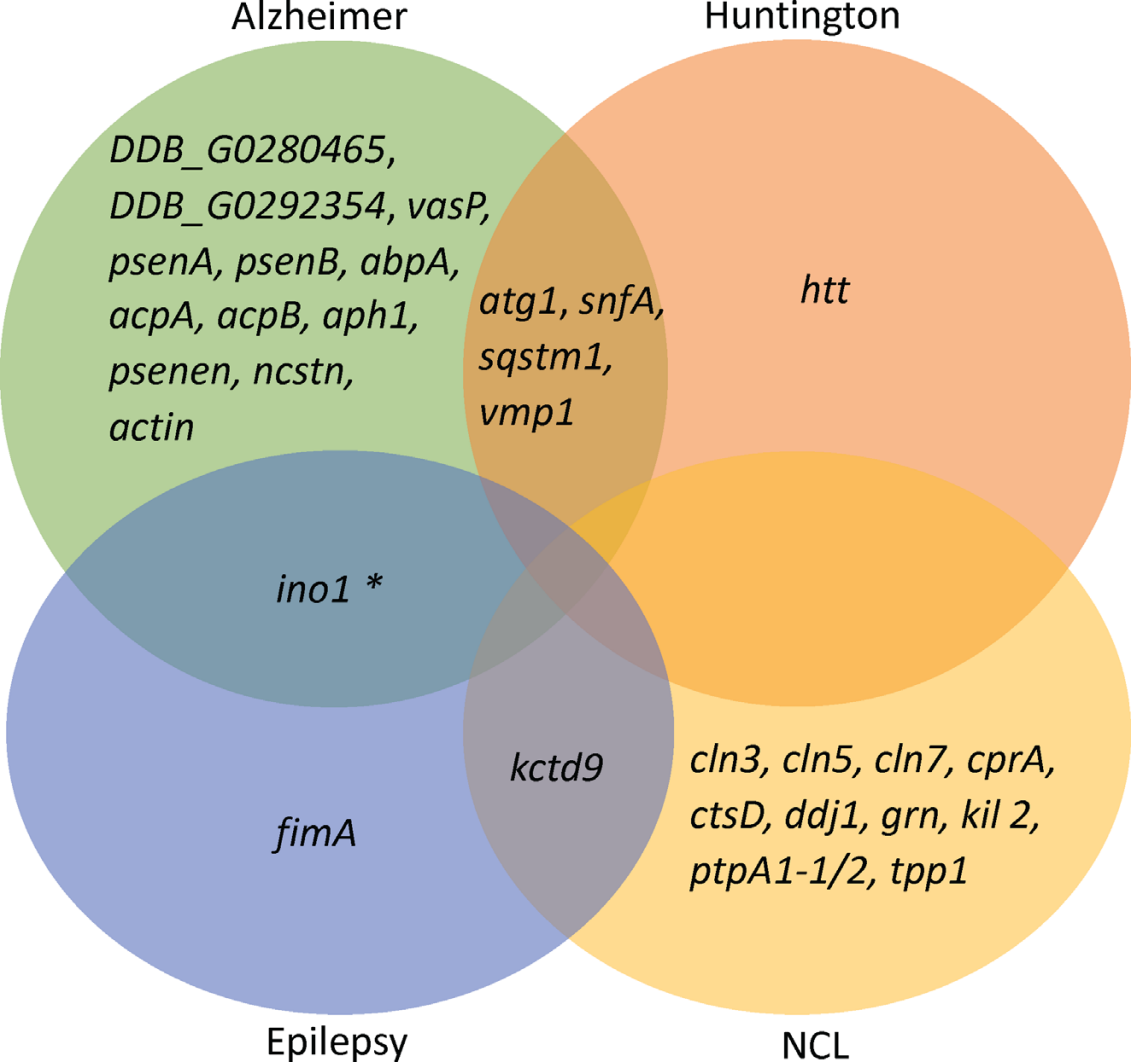
*D. discoideum* genes implicated in neurological disorders. This Venn diagram includes some of the most interesting genes in *D. discoideum* for the study of neurological diseases in humans including Alzheimer, Huntington, epilepsy, neuronal ceroid lipofuscinosis and bipolar disorder. *ino1 is also related with bipolar disease which is not represented in the figure.

*D. discoideum* is also a good model for the study of some neurological disorders including Alzheimer, Huntington, epilepsy, bipolar disorder or neuronal ceroid lipofuscinoses (Myre, 2012; Frej *et al*., 2017; McLaren *et al*., 2019). One of the biggest advantages of using *D. discoideum* in neuronal disorders is that unlike mammalian models where some of these genes are essential for embryogenesis such as *HTT*, presenilins (*psenA* and *psenB*) or amyloid beta peptide, in *D. discoideum* they are not and therefore, they can be mutated (Myre, 2012). Also, the genes related with the neural pathology in mammals do not exist in some cases in other eukaryotic models but appears in *D. discoideum*, including but not limited to genes responsible for the neuronal ceroid lipofuscinosis (NCL) (McLaren *et al*., 2019). Main *Dictyostelium* proteins studied in neurological disorders with human orthologues are listed in Figure 3 and Table S3.

Alzheimer is a neurodegenerative disorder that causes dementia (Myre, 2012). In Alzheimer, Hirano bodies, amyloid plaques and neurofibrillary tangles are the hallmarks of this disease. The Hirano bodies are an aggregate of actin filaments with actin‐interacting proteins, whose function is unknown in the biology of the disease (Carnell and Insall, 2011). Interestingly, *D. discoideum* cells can synthetize very similar aggregates to Hirano bodies (Maselli *et al*., 2003; Carnell and Insall, 2011). The main component of Hirano bodies that were found in *D. discoideum* cells correspond to a 34 kDa actin cross‐linking protein with an aberrant C‐terminal portion (Carnell and Insall, 2011; Myre, 2012) (Fig. 3). Researchers have found that some cases of the disorder can result from mutations in the *APP*, *PSEN1* or *PSEN2* genes (Sherrington *et al*., 1995). *APP* encodes amyloid precursor protein, whereas *PSEN1* and *PSEN2* encode presenilin 1 and 2 respectively (Fig. 3). When any of these genes is altered, large amounts of a toxic protein fragment called amyloid beta peptide are produced in the brain (Myre, 2012). This peptide can build up in the brain to form clumps called amyloid plaques (Myre, 2012). Although *APP* is not present in *D. discoideum*, cells expressing mammalian *APP* were able to process it and form Aβ40/Aβ42, the peptides that cause the Alzheimer’s disease in humans (Myre, 2012). On the other hand, presenilin protein in *Dictyostelium*, as in mammals, is a component of the γ‐secretase complex (*aph1*, *psenen* and *ncstn*) and is essential in *Dictyostelium* differentiation (Myre, 2012). Myo‐inositol is an abundant carbocyclic sugar in brain and other mammalian tissues where it plays an important role as the structural basis for a number of secondary messengers in eukaryotic cells, mediating cell signal transduction in response to a variety of hormones, neurotransmitters and growth factors. In addition, inositol serves as an important component of the structural lipids phosphatidylinositol (PI) and the phosphatidylinositol phosphate (PIP) lipids (Frej *et al*., 2017). Myo‐inositol has been largely studied in human cells, as well as in *Dictyostelium* (Frej *et al*., 2017). Some studies in *Dictyostelium* suggested that INO1 (*ino1*), a key enzyme in myo‐inositol biosynthesis pathway, is responsible for metabolic changes resulting in elevated protein degradation, glucose breakdown and high levels of amino acids (Frej *et al*., 2016). Other *Dictyostelium* proteins implicated in Alzheimer's disease are tau‐tubulin kinase orthologue (*DDB_G0292354*) (Manning *et al*., 2002) and 3‐hydroxyacyl‐CoA dehydrogenase type‐2 (*DDB_G0280465*) (Fig. 3).

Huntington’s disease is a progressive neurodegenerative disorder with many consequences such as motor, cognitive and behavioural disturbances. It is caused by mutations in the *HTT* gene coding for a protein called huntingtin which plays an important role in neurons in the brain and is essential for normal development before birth (Bates, 2005). The hallmark of Huntington’s disease is the high repetition of a CAG trinucleotide leading to the expansion of the *HTT* gene, that confers a gain‐of‐function property (Bozzaro, 2013). *Dictyostelium* represents a good model to study this disease since it has a huntingtin human orthologue whose mutation or deficiency is not lethal for the cells (Myre, 2012). *Dictyostelium htt* mutant cells show pleiotropic defects such as reduced cell–cell and cell‐substratum adhesion, delayed development, a strong cellular sensitivity to osmotic stress, cytoskeletal defects, chemotaxis defects and regulate cell fate during development (Thompson *et al*., 2014; Bhadoriya *et al*., 2019). These pleiotropic defects are consistent with the *in vitro* observations using human cells from Huntington’s disease patients (Bozzaro, 2013). Alzheimer and Huntington’s disease patients were found to have increased levels of AMPK (Annesley and Fisher, 2009). In response to reduction of intracellular ATP levels, AMPK activates energy‐producing pathways and inhibits energy‐consuming processes, as well as cell growth and proliferation (Annesley *et al*., 2014). As mentioned above, AMPK is conserved in *D. discoideum* and it is important as a central regulator of energy production in the cells (Fig. 3).

Another neurological pathology where *D. discoideum* has been very useful is in epilepsy, although the genetics of epilepsy are complex and not completely understood. Seizures is the main hallmark of this neurological disorder, and as in Alzheimer, the myo‐inositol balance is critical in humans (Wellard *et al*., 2003; Frej *et al*., 2017). Apart from myo‐inositol, some studies in *D. discoideum* show that the phosphoinositide PIP₃ is reduced during seizure activity (Chang *et al*., 2014; Frej *et al*., 2017). PIP₃ regulates voltage‐gated channel (Viard *et al*., 2004), neuronal excitability (MacGregor *et al*., 2002) and insertion of ion channels into synaptic plasma membranes (Lhuillier and Dryer, 2002). PIP₂ is also important in the develop of seizures in the DOORS syndrome, a disorder involving multiple abnormalities, caused by mutations in the *TBC1D24* gene (Fischer *et al*., 2016; Frej *et al*., 2017). Another key player protein is calmodulin (*calA*), observed in human cells, not only related to heart arrhythmias, but also to epilepsy and delayed neurodevelopment (O'day *et al*., 2020). *Dictyostelium* represents a good model for the study of CalA mutations thanks to its highly conserved structure, haploid genome and the possibility to obtain numerous mutations (O'day *et al*., 2020).

As mentioned above, *D. discoideum* has been very useful to study neuronal ceroid lipofuscinosis (NCL), a group of inherited progressive degenerative brain diseases characterized clinically by a decline of mental and other capacities, epilepsy and vision loss through retinal degeneration (McLaren *et al*., 2019). Related to this pathology, there are 13 genes that when mutated are known to cause the disorder (Fig. 3), 11 out of these 13 genes are conserved in *D. discoideum*. These 11 genes are *Ppt1* (*CLN1* orthologue) (Phillips and Gomer, 2015), *Tpp1* (*CLN2* orthologue) (Phillips and Gomer, 2015), *CtsD* (*CLN10* orthologue) (Ashworth and Quance, 1972) and *CprA* (*CLN13* orthologue) which encode lysosomal enzymes, *Ddj1* (*CLN4* orthologue) and *Kctd9* (*CLN14* orthologue) which are membrane proteins, *Cln5* (*CLN5* orthologue) a soluble lysosomal protein, *Grn* (*CLN11* orthologue) a granulin domain‐containing protein and *Cln3* (*CLN3* orthologue) (Huber *et al*., 2014), *Mfsd8* (*CLN7* orthologue) and *Kil2* (*CLN12* orthologue) coding for transmembrane proteins that localize in different organelles (Huber, 2016). Interestingly, *Ppt1*, *Tpp1*, *CLN3* (Huber *et al*., 2014), *CLN5* and *CtsD* have been studied in *Dictyostelium* (Huber, 2016; McLaren *et al*., 2019). Promising results are expected using *D. discoideum* as a model for the study of NCL (Huber, 2016).

Bipolar disorder is a neuropsychiatric disorder where the patients have severe mood, energy and behaviour swings (Frej *et al*., 2017). Very little is known about the genetics of bipolar disorder, although some of the genetic changes associated with bipolar disorder have also been found in people with other common mental health disorders, such as schizophrenia. Understanding the genetics of bipolar disorder and other forms of mental illness is an active area of research. Both, the myo‐inositol imbalance and autophagy are essential in the course of this pathology. Indeed, the inositol changes are the target of the actual treatment of the disease (Frej *et al*., 2017). The way that VPA and lithium (the treatment) produce a reduction of myo‐inositol has been studied in many model organisms including *D. discoideum*. Studies using *Dictyostelium* show that these treatments cause an intracellular reduction of InsP₃ (Eickholt *et al*., 2005) and increase the INO1 transcription (Vaden *et al*., 2001). VPA also causes a reduction of phosphoinositides like PIP₂ (Xu *et al*., 2007) while lithium causes a suppression of PIP₃‐mediated signalling (King *et al*., 2009; Frej *et al*., 2017). Hence, the dysregulation of phosphoinositides and inositol are linked with the pathology (Frej *et al*., 2017).

## *Dictyostelium discoideum* as a model organism in autophagy/phagocytosis

Autophagy is a fast‐moving field with an enormous impact on human health and disease which has benefited from the use of *D. discoideum*. *D. discoideum* has shed light on the mechanisms that regulate autophagosome formation and contributed significantly to the study of autophagy‐related pathologies (Mesquita *et al*., 2017). Importantly, autophagy is a process associated with the infection of pathogens such as *L. pneumophila* and *S. thyphimurium* and involved in neurodegenerative disorders and cancer. About 17 proteins related to autophagy (the so‐called ATG proteins) have been annotated in *D. discoideum* (Chisholm, 2006). *D. discoideum* ATG mutants result in phenotypes with reduced survival under nitrogen starvation, impaired endocytosis and growth, aberrant morphogenesis and defective spore differentiation (Annesley *et al*., 2014). Among the proteins mediating autophagy ATG8 (*atg8*), ATG9 (*atg9*) and ATG16 function on phagocytosis (Bozzaro and Eichinger, 2011). In particular, ATG8 was suggested as a great marker of the autophagy linked with autophagosome–lysosome fusion (Meßling *et al*., 2017). ATG *Dictyostelium* proteins and other key proteins related to autophagy/phagocytosis are listed in Tables 3 and S4. *D. discoideum* has also been used to study autophagy in the elimination of pathogens such as *S. aureus, S. enterica, F. noatunensis* and *M. marinum* (Mesquita *et al*., 2017). Some of the studies are related with the process called ejection, that leads to the escape of the bacteria. Several proteins are involved in the ejection process including but not limited to ATG8, ATG18 (*atg18*) and Sqstm1 (*sqstm1*) (Mesquita *et al*., 2017). As mentioned above, ATG9 and ATG16 play a role in the uptake (Leoni Swart *et al*., 2018).

**Table 3:** *D. discoideum* proteins involved in pathologies caused by autophagy and phagocytosis defects. A total of 32 proteins have been identified. The proteins are grouped based on their biological function, including autophagy, endosomal transport, multivesicular body organization, vacuole organization, multivesicular body assembly and vacuolar transport.

Gene name (*Dictyostelium*)	UniProt (*Dictyostelium*)
Autophagy	
*atg1*	Q86CS2
*cdcD*	P90532
*psenA*	Q54ET2
*psenB*	Q54DE8
*iplA*	Q9NA13
Multivesicular body assembly	
*atg5*	Q54GT9
*atg6*	Q55CC5
*atg7*	Q86CR9
*atg9*	Q54NA3
*vps20*	Q54KZ4
*vps22*	Q54RC4
*vps24*	Q54P63
*vps25*	Q55GD9
*vps2A*	Q54GK9
*vps2B*	Q54DB1
*vps36*	Q54T18
*vps60*	Q54JK4
*ugpB*	Q54YZ0
*glcS*	Q55GH4
Multivesicular body organization	
*sqstm1*	Q55CE3
*wshA*	Q54CK9
*talB*	Q54K81
Endosomal transport	
*tipC*	Q55FG3
*vps35*	Q54C24
*vps4*	Q54PT2
Vacuole organization	
*vmp1*	Q54NL4
Vacuolar transport	
*tsg101*	Q54LJ3
*vps13A*	Q54LB8
*vps13B*	Q555C6
*vps13D*	Q54LN2
*vps28*	Q54NF1
*vps37*	Q55DV8

There is evidence of a possible relation between Alzheimer, Huntington and Parkinson diseases, and autophagy dysfunction, that leads to accumulate aberrant organelles and proteins (Mesquita *et al*., 2017). Some candidates include Vmp1 (*vmp1*), Sqtm1, ATG5 (*atg5*) or ATG1, which are involved in protein degradation, or ATG8, ATG5 and ATG1 involved in Hirano bodies‐like aggregates degradation in Alzheimer (Mesquita *et al*., 2017). Regarding Hirano bodies present in Alzheimer, it has been shown that *Dictyostelium* can degrade the Hirano bodies‐like aggregates by autophagy and the proteasome (Mesquita *et al*., 2017) In neurodegenerative disorders, other protein related with autophagy dysfunction is the VPS13 (*vps13A*, *vps13B* and *vps13D*) involved in Parkinson’s disease and Chorea‐acanthocytosis (Mesquita *et al*., 2017). Also, the gene *KIAA0196/Strumpellin* which encodes a component of the WASH complex is related with the autosomal dominant hereditary spastic paraplegia in humans (Mesquita *et al*., 2017). The CdcD protein (*cdcD*), orthologue of VCP/p97 in humans, is related with cell death by autophagy in the presence of aberrant mitochondria and has been linked to IBMPFD (inclusion body myopathy with early onset Paget's disease of bone and frontotemporal dementia), HSP (hereditary spastic paraplegia), and a form of ALS (amyotrophic lateral sclerosis) (Annesley *et al*., 2014).

The process that causes cell death by autophagy is called autophagic cell death (ACD), and it is related with tumour suppression and neurological disorders as a consequence of psychological stress (Jung *et al*., 2020). Interestingly, this process was also observed in *D. discoideum* (Jung *et al*., 2020). This process starts in the stalk cells during starvation and finalize with the presence of differentiation factor DIF‐1 for cell death induction (Jung *et al*., 2020). However, it was found that in *Dictyostelium* this process is prevented when some genes are mutated including *atg1*, *iplA*, *talB*, *ugpB* and *glcS* (Jung *et al*., 2020).

## Mitochondrial syndromes

Mitochondrial diseases are genetic disorders that occur when mitochondria fail to produce enough energy for proper body function. Different diseases may arise including but not limited to metabolic strokes, seizures, cardiomyopathy, arrhythmias, developmental and cognitive disabilities (Barth *et al*., 2007). Some mitochondrial syndromes are closely related to neurodegenerative diseases such as Huntington and Alzheimer (Annesley and Fisher, 2009), whereas other mitochondrial syndromes are related with diabetes, myopathy, kidney disease, blindness or deafness (Pearce *et al*., 2019) The mitochondrial genome of *Dictyostelium* has 55,564 base pairs, its circular and encodes 33 proteins (Barth *et al*., 2007). It also contains six ORFs, two ribosomal RNA genes and 18 transfer RNA genes (Barth *et al*., 2007). The proteins are mainly involved in respiration and translation (Barth *et al*., 2007). There are some important similarities between human and *Dictyostelium* mitochondrial DNA including the main oxidative phosphorylation pathway (Pearce *et al*., 2019). Indeed, important proteins implicated in mitochondrial syndromes have been studied in *D. discoideum* (Table 4 and Table S5).

**Table 4:** Main *D. discoideum* proteins involved in mitochondrial disorders. Most studied proteins are encoded in the nuclear genome, whereas some other proteins are encoded in the mitochondrial genome.

Mitochondrial proteins encoded in nuclear genome			
Gene name (*Dictyostelium*)	UniProt (*Dictyostelium*)	Gene name (*Dictyostelium*)	UniProt (*Dictyostelium*)
*abpC*	P13466	*ndufs8*	Q86K57
*cap*	P54654	*ndufv1*	Q54I90
*cluA*	O15818	*ndufv2*	Q54F10
*DDB_G0267514*	Q55GU0	*piaA*	O77203
*DDB_G0267552*	Q55GR1	*pkbA*	P54644
*DDB_G0275973*	Q552K6	*raptor*	Q55BR7
*DDB_G0279405*	Q54WW7	*rasD, gefE, NS gefL*	P03967, Q8IS18, B0M0P8
*DDB_G0291852*	Q54E48	*rblA*	Q54FX2
*DDB_G0292722*	Q54CZ9	*regA*	Q23917
*hspA*	Q54J97	*ripA*	C7G030
*lkb1*	Q54WJ0	*rps4*	P51405
*lst8*	Q54D08	*snfA*	Q54YF2
*midA*	Q54S83	*tor*	Q86C65
*ndufaf5*	Q54JW0	*trap1*	Q86L04
*ndufs4*	Q8T1V6	*GcvH1*	Q54JV8
*ndufs7*	Q54NI6	*DDB_G0281081*	Q54UH1

Proteins encoded in mitochondrial genome	
Gene name (*Dictyostelium*)	UniProt (*Dictyostelium*)
*atp6*	Q27559
*cox3*	O21049
*nad1*	Q37313
*nad11*	Q34312
*nad2*	O21048
*nad3*	Q37312
*nad4*	O21047
*nad5*	Q34313
*nad7*	Q23883
*nad9*	P22237

Many mitochondrial syndromes have been related with nuclear encoded proteins that exert their role in mitochondria, mainly AMPK, which overactivation is neurotoxic and it is related with AICA‐ribosiduria, amyotrophic lateral sclerosis (ALS), Alzheimer, Huntington and Parkinson (Annesley and Fisher, 2009; Annesley *et al*., 2014). In *D. discoideum,* the kinases involved in AMPK activation are LKB1 (*lkb1*), TAK1 (*DDB_G0267514*) and CaMKK2 (*DDB_G0279405*) (Annesley *et al*., 2014). The mitochondrial chaperonin 60 protein (Cpn60, *hspA*) in *D. discoideum*, is not only related with neurological disorder but also causes developmental diseases, respiratory enzyme deficiencies and early infancy death (Barth *et al*., 2007). Complex I dysfunction, with various factors present in *D. discoideum*, causes Leigh syndrome, Parkinson and Alzheimer (Francione *et al*., 2011). *D. discoideum* was also used to study the mitochondria glycine cleavage system, GCVH1, orthologue of the human GCSH, which is involved in epilepsy (Perry *et al*., 2020). Thanks to *D. discoideum* we now know that CBD (Cannabidiol) could be a good treatment for this pathology (Perry *et al*., 2020). *D. discoideum htrA,* orthologue of the human protein HTRA2, was found to play a proteolytic role in mitochondria and its function was related with autosomal dominant late‐onset Parkinson’s disease (Chen *et al*., 2018). The most common *D. discoideum* phenotypes related to mitochondrial syndromes are impaired phototaxis and thermotaxis, aberrant multicellular morphogenesis, impaired aggregation and growth and altered phagocytosis (Barth *et al*., 2007). Heteroplasmy also happens in *D. discoideum* and results in severe phenotypes depending on the number of mitochondrial DNA copies altered (Annesley and Fisher, 2009).

## Pharmacological treatments

*Dictyostelium discoideum*, as a pharmacological model, provides useful insight into the cellular and molecular functions of both therapeutic drugs and pharmacologically active natural products (Schaf *et al*., 2019). In particular, the haploid genome of *D. discoideum* and its amenability to genetic manipulation has helped with the identification of specific genes involved in some pharmacological treatments. One of the most studied drugs in *Dictyostelium* is valproic acid (VPA). VPA is the most highly prescribed epilepsy treatment worldwide, also used to prevent bipolar disorder and migraine, since it has been demonstrated to have neuroprotective effects in neurodegenerative conditions (Terbach *et al*., 2011). Some of the VPA targets identified in *Dictyostelium* are InsP₃, InsP₂ (provoking a reduction) and PIP₃ (protecting against its reduction) (Frej *et al*., 2017), INO1 (raising its expression) (Frej *et al*., 2017), DGKA (Kelly *et al*., 2018), solute carrier 4 bicarbonate transporter (SLC4) (Terbach *et al*., 2011), histone deacetylase (Cunliffe *et al*., 2015) and phospholipase A2 (Elphick *et al*., 2012). Treatments for the control of seizures and bipolar disorder studied in *D. discoideum* include VPA (Frej *et al*., 2017), lithium (Frej *et al*., 2017) and medium fatty acid (such as decanoic acid)(Cunliffe *et al*., 2015), all of them act regulating phosphoinositides levels. Lithium and decanoic acid are also involved in DGKA and in InsP₃ reduction as seen for VPA (Cunliffe *et al*., 2015; Frej *et al*., 2017; Kelly *et al*., 2018).

Due to its activity as a regulator of cell growth, cell death and anti‐/pro‐oxidant, curcumin has been investigated in *Dictyostelium* as a treatment for Alzheimer, Parkinson, multiple sclerosis cardiovascular diseases, cancer, allergy, asthma, rheumatoid arthritism, diabetes and inflammation (Cocorocchio *et al*., 2018). *D. discoideum* has allowed to discover two curcumin targets, phosphatase 2A regulatory subunit (*psrA*) and presenilin‐1 (*PsenB*). Both proteins are conserved in human, PP2A and PS1 respectively. The orthologue for the phosphatase 2A regulatory subunit is the subunit B56 of the PP2A protein, involved in many functions such as cell proliferation, signal transduction, apoptosis and related with some cancers (Cho and Xu, 2007). Presenilin‐1 human orthologue (PS1) is involved in the APP cleavage and it has a key role in the develop of Alzheimer’s disease (Cocorocchio *et al*., 2018). Curcumin acts maintaining PP2A subunit B, leading to Tau dephosphorylation and GSK3β inhibition leading to growth arrest in some cancers (Cocorocchio *et al*., 2018).

*Dictyostelium* did not respond to salty, sour, umami or sweet tasting compounds; however, cells rapidly responded to bitter tastants (Cocorocchio *et al*., 2016). Tastants are taste‐provoking chemical molecules that are dissolved in ingested liquids or saliva to stimulate the sense of taste. *Dictyostelium* showed varying responses to the bitter tastants, providing a suitable model for early prediction of bitterness for novel tastants and drugs (Cocorocchio *et al*., 2016). For instance, a novel human receptor involved in bitter tastant detection was identified using *Dictyostelium discoideum* (Robery *et al*., 2013). *Dictyostelium* is a good model to study the bitter tastant and could replace the actual model which is the rat *in vivo* brief access taste aversion (BATA). Other approaches include the study of naringenin and aminobisphosphonates (Misty *et al*., 2006; Grove *et al*., 2010; Waheed *et al*., 2014). The action of naringenin, a dietary flavonoid with antiproliferative and chemopreventive actions of carcinogenesis, was investigated as a potential new therapeutic agent in autosomal dominant polycystic kidney disease (Waheed *et al*., 2014). On the other hand, *Dictyostelium* allowed to identify the enzyme farnesyl diphosphate synthase (FDP) as the target of aminobisphosphonate bone resorption inhibitors in mammalian osteoclasts (Sugden *et al*., 2005).

## Conclusions and perspectives

**Fig. 4:**
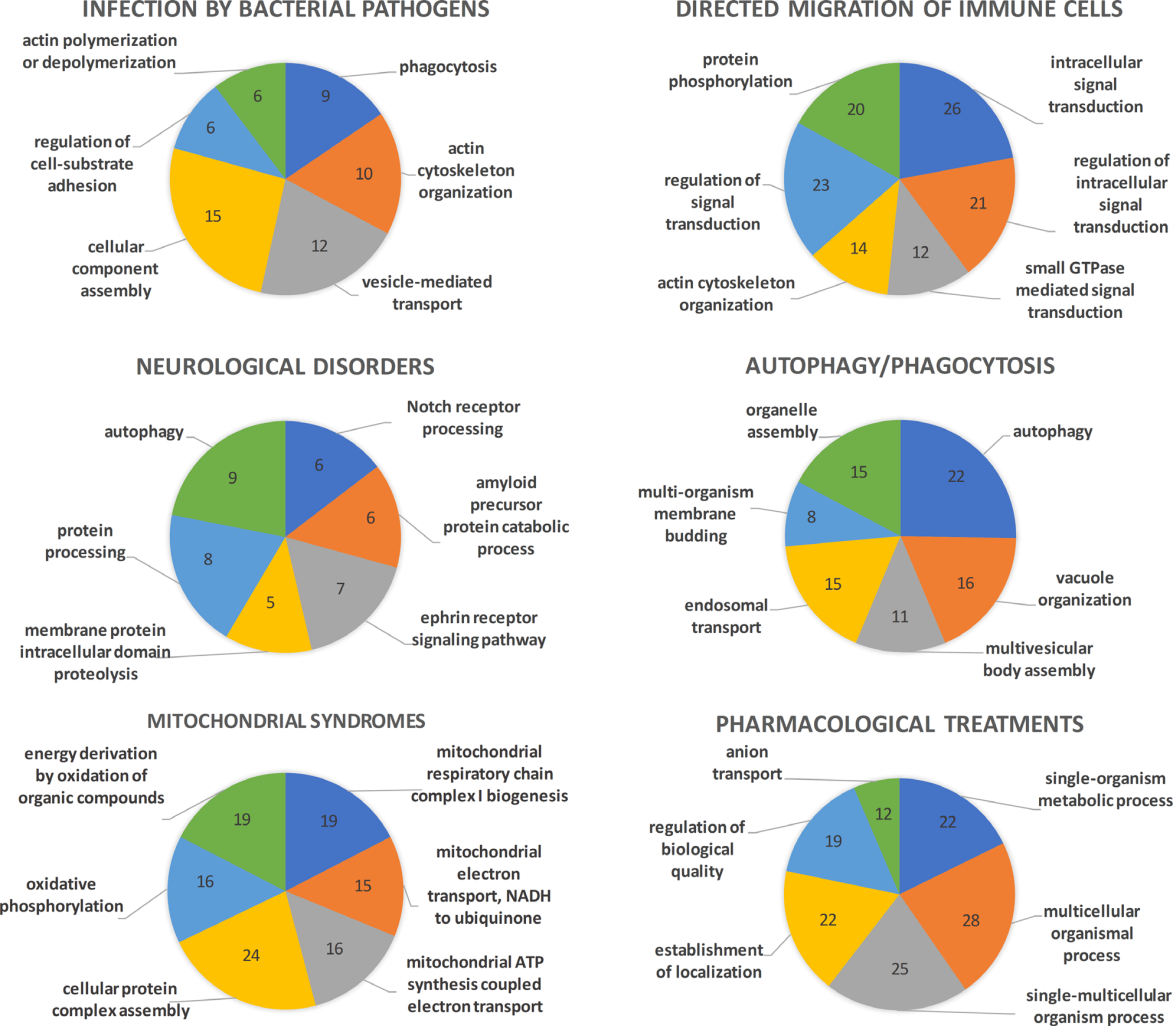
Biological implications of *Dictyostelium discoideum* proteins according to the different fields reported in this review. The six most significant GO terms for biological processes (by *P*‐value, and avoiding redundancy) are represented based on their functional annotation of human Uniprot IDs with DAVID 6.7 (Huang *et al*., 2009, 2009,2009, 2009). Numbers indicate the proteins associated with each biological function. For further detailed information, please refer to Table S6.

*D. discoideum* represents a good model to study different pathologies with high incidence in human health. So far, *D. discoideum* has proven to be a suitable model for the study of neurological diseases including but not limited to Alzheimer, epilepsy, bipolar disease, NCL and Huntington. Indeed, *D. discoideum* was used as an advantageous model for pharmacogenetic research in both epilepsy and bipolar disease. Besides, *D. discoideum* was used as a research model in major findings related with other pathologies including Wiskott–Aldrich and Shwachman–Diamond syndromes, in autophagy and mitochondrial syndromes, neurodegenerative diseases and cancer. *D. discoideum* can be infected by *Legionella* (and many other pathogens) and this information provided insight into the proteins involved in the process in order to eventually understand better how to fight these human pathogens. *D. discoideum* also has de ability to chemotax, like human leucocytes or tumour cells, and that is why it has been chosen as the key model organism for the study of eukaryotic chemotaxis. *D. discoideum* is an exceptional model organism to study a wide range of neurological disorders, many of them characterized by altered mitochondrial dynamics, structure and/or function, proven *D. discoideum* as a mitochondrial disease system. Thanks to *D. discoideum* it was possible to identify and study proteins involved in those neurological disorders, in part because *D. discoideum* cells do not exhibit variation in symptoms, thus simplifying the study on mitochondrial diseases. Studied proteins related to the aforementioned diseases are involved in actin cytoskeleton, endocytosis, transport, metabolism and signalling pathways including but not limited to RAS and Notch (Fig. 4). The analysis of the biological function of the proteins studied in *D. discoideum*, based on their human orthologues, evidences the cellular mechanisms that can be targeted using this model organism (Fig. 4). A total of 27 *D. discoideum* proteins are related to infection by bacterial pathogens and correspond mainly to cellular adhesion to substrate and component assembly, actin‐related mechanisms and vesicle transport and phagocytosis. From the 28 *D. discoideum* proteins related to directed migration of immune cells, most are involved in processes of signal transduction including small GTPase mediation, protein phosphorylation and actin cytoskeleton organization. In neurological disorders, up to 33 *D. discoideum* proteins are related to the Notch and ephrin receptors, amyloid precursor metabolism, processing and proteolysis of proteins and autophagy. In autophagy and phagocytosis, with 60 *D. discoideum* proteins, the main related processes include but are not limited to vacuolar, endosomal and multivesicular management, organelle and vesicle assembly, and membrane budding. Related to mitochondrial syndromes, the studied 37 *D. discoideum* proteins are implicated in the respiratory chain, ATP synthesis and NADH enzymatic reactions. Finally, pharmacological treatment studies have been done with 45 *D. discoideum* proteins that tackle both single and multicellular processes with a focus on transport and localization, anion transport and biological quality. Further information on the biological processes and the associated proteins can be found in Table S6. Many of these discoveries need to be done in mammalian cell lines, thus enabling to corroborate the results obtained. The use of *Dictyostelium* addresses the development of the principles of the 3Rs in research (replacement, refinement and reduction), to reduce the reliance on the use of animal tissue and whole‐animal experiments (Otto *et al*., 2016), which might lead to an increasing number of studies using this social amoeba as a biomedical model in the upcoming years.

## Conflict of interest

The authors declare no conflict of interest.

## Supporting information


**Table S1.** *Dictyostelium* proteins involved in *Legionella pneumophila* infection.

**Table S2.** *Dictyostelium* proteins involved in directed cell migration.

**Table S3.** *Dictyostelium* proteins involved in neurological disorders.

**Table S4.** *Dictyostelium* proteins involved in autophagy/phagocytosis.

**Table S5.** *Dictyostelium* proteins involved in mitochondrial syndromes.

**Table S6.** GO Term analysis for biological processes associated to the proteins studied in *Dictyostelium*.
